# Predicting Chronic Liver Disease Severity by Liver and Splenic Extracellular Volume Fraction Derived from spectral-CT

**DOI:** 10.2174/0115734056396041250728133515

**Published:** 2025-08-04

**Authors:** Yiming Yang, Zhiyuan Chen, Dongjing Zhou, Mengya Guo, Yan Qi, Mengqi Yu, Yupin Liu

**Affiliations:** 1 Department of Radiology, The Second Affiliated Hospital of Guangzhou University of Chinese Medicine, Guangzhou, China; 2 Department of Imaging Research Center, GE HealthCare, Guangzhou, China

**Keywords:** Chronic liver disease, Severity stratification, Spectral computed tomography, Liver, Spleen, Extracellular volume fraction

## Abstract

**Introduction:**

To evaluate the effectiveness of spectral-CT in assessing the severity of liver diseases in patients with chronic liver disease (CLD).

**Methods:**

A total of 148 CLD patients who underwent spectral-CT were retrospectively recruited, including 40 non-advanced CLD (non-ACLD), 74 compensated ACLD (cACLD), and 34 decompensated ACLD (dACLD). Iodine concentrations in the liver and spleen were assessed on iodine (water) images during the equilibrium phase, which allowed for the calculation of liver and splenic extracellular volume fractions (ECV). We determined the total liver volume, liver segmental volume ratio, and splenic volume from portal phase images. Moreover, established non-invasive tests were also collected. Areas under receiver operating characteristic curve (AUCs) were employed to evaluate the diagnostic performance of CT parameters and non-invasive tests in predicting CLD severity. Additionally, we analyzed the correlations between CT parameters and non-invasive tests.

**Results:**

The spleen volume demonstrated the highest AUC (0.815, P<0.001) for distinguishing between non-ACLD and cALCD. Child-Pugh score exhibited the highest AUC (0.948, P<0.001) for distinguishing cALCD and dACLD. Splenic ECV exhibited the highest AUC (0.853, P<0.001) for distinguishing non-ALCD and ACLD. In contrast, the liver ECV showed strong correlations with the Fibrosis-4 Index (r=0.653, p<0.001) and the Aminotransferase-to-Platelet Ratio Index (r=0.607, p<0.001), while spleen ECV correlated more strongly with the Child-Pugh score (r=0.719, p<0.001) and the Albumin-Bilirubin Index (r=0.742, p<0.001).

**Discussion:**

Liver and splenic ECV can effectively reflect the dynamic progression of CLD and correlate well with non-invasive tests in these patients.

**Conclusion:**

Spectral-CT liver and splenic ECV could serve as non-invasive imaging biomarkers for severity stratification.

## 1 INTRODUCTION

For patients with chronic liver disease (CLD), severity classification is crucial for identifying patients with poor prognosis and tailored treatment regimens [1]. The BAVENO VII consensus proposed the concept of compensated advanced chronic liver disease (cACLD) based on liver stiffness measurement (LSM) to better reflect the spectrum of severe fibrosis and cirrhosis, while decompensated advanced chronic liver disease (dACLD) is defined based on a history of liver function decompensation (moderate/severe ascites, variceal hemorrhage, or hepatic encephalopathy) [2]. Given that the various disease stages are associated with differ markedly in prognosis, patients in different stages have different diagnostic and therapeutic needs [2, 3]. However, due to the need for specific hardware, LSM is not available in all medical institutions.

Currently, various non-invasive tests are applied to assess the severity of patients with CLD. For example, the Fibrosis index based on the 4 factors (FIB-4) and the Aminotransferase-to-platelet ratio index (APRI) stand as pivotal markers in gauging the progression of liver fibrosis [4]. Beyond these indices, clinical/laboratory indicators such as such Child-Pugh classification, Albumin-Bilirubin (ALBI) grade have long been used for stratifying CLD patients according to the severity of liver function impairment [5]. But the laboratory-indicators-based non-invasive tests can be influenced by extrahepatic factors and may lead to an under or over-estimate of CLD severity in some patients [6-8]. To overcome these limitations, a combination of imaging examinations and clinical/laboratory parameters may provide a more comprehensive understanding of the process of CLD.

As is commonly known, CLD patients often undergo CT (Computed Tomography) scans to monitor disease progression and identify malignancies. Since CLD has significant differences in the CT structural features of their liver and spleen, such as relative hypertrophy of the caudate and left lateral lobes, and splenomegaly [1, 9]. Therefore, some studies have indicated that CT volume measurement can serve as a marker of liver fibrosis and cirrhosis. Feng *et al* [10] found that cirrhotic patients had lower average total liver volume (TLV) and higher average splenic volume (SV) than those in the healthy group. Tago *et al*’s [11] study showed that standardized SV is superior to standardized TLV and the volume ratio of Couinaud segments I-III to segments IV-VIII (liver segment volume ratio: LSVR) in estimating liver fibrosis grades. However, further validation is needed to prove its diagnostic value for assessing CLD severity.

Extracellular volume fraction (ECV) is an index calculated by measuring Hounsfield Units (HU) on pre-contrast and post-contrast equilibrium phase CT scans, which is used to evaluate the iodine accumulated in the extracellular space [12]. Spectral-CT is a kind of is an imaging technique based on X-ray absorption of two different energies, which enables the direct measurement of iodine concentration in the extracellular space through a process known as “material decomposition” [13, 14]. The study by Bottari *et al* [15]reveals that the liver ECV measured by dual-energy CT is a feasible and non-invasive method for evaluating cirrhosis, with an area under the curve (AUC) of 0.88. Kokubo *et al* [16] studied the correlation between liver and splenic ECV and non-invasive tests, manifesting the capability of ECV in evaluating liver function. However, the role of spectral-CT ECV in the severity stratification for patients with CLD (including non-advanced chronic liver disease [non-ACLD], cACLD, and dACLD) is not yet clear.

The purpose of this study is to investigate spectral-CT liver and splenic ECV and CT volume-related parameters in predicting the severity of patients with CLD, and to compare their performance against established non-invasive tests.

## 2 MATERIALS AND METHODS

### 2.1 Subjects

This retrospective study was conducted in accordance with the Declaration of Helsinki and approved by the review board of The Second Affiliated Hospital of Guangzhou University of Chinese Medicine (ZE-2024-311-01). 197 established diagnosis CLD patients (including patients with hepatitis B virus, hepatitis C virus, alcoholic liver disease, and autoimmune liver disease) who underwent multiphase 256-slice spectral-CT were identified between March 2023 and June 2024.

Exclusion criteria were as follows: (1) current or prior malignancy, (2) post-hepatic surgery, (3) previous transcatheter splenic arterial embolization or splenectomy, (4) history of transjugular intrahepatic portosystemic shunt placement, and (5) patients with missing data of the clinical or laboratory parameters.

**Fig. (1):**
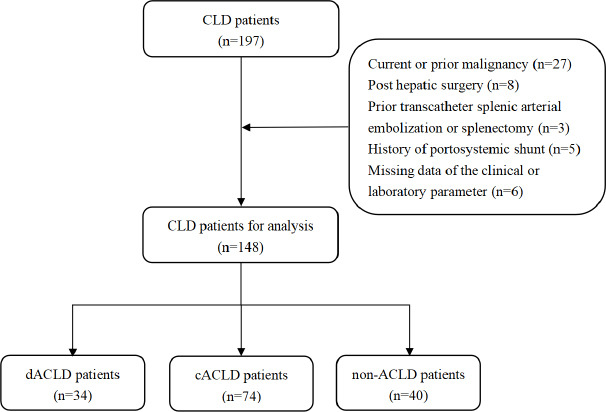
Patient flowchart.

After screening, 148 patients with CLD were included in the study, with 34 patients in the dACLD group, 74 patients in the cACLD group, and 40 patients in the non-ACLD group (Fig. **1**).

### 2.2 Staging of CLD

Based on the liver stiffness measurement (LSM; FibroScan, Echosens, France), radiological features, and history of hepatic decompensation, patients were classified as having non-ACLD (LSM<10 kPa), cACLD (cirrhosis radiological features [nodular liver, splenomegaly or varices] or LSM≥10 kPa), and dACLD (history of moderate/severe ascites, variceal hemorrhage or hepatic encephalopathy) [1]. LSM and the history of variceal hemorrhage or hepatic encephalopathy were retrospectively collected from the objective records within the medical record system. Nodular liver, splenomegaly, varices, and moderate/severe ascites were evaluated by two radiologists with 4 and 7 years of working experience, respectively. When there were inter-observer discrepancies, a radiologist with 12 years of working experience was consulted to reach a consensus.

### 2.3 Clinical Data

Blood test data (hematocrit [Hct], total bilirubin [TB], albumin [Alb], prothrombin time [PT], aspartate aminotransferase [AST], alanine aminotransferase [ALT], platelet count [PLT]), were obtained from the electronic medical records.

Based on clinical/laboratory parameters, Child-Pugh score, ALBI, FIB-4, and APRI were calculated using the standard formulas. ALBI = log10 (TB [µmol/L]) × 0.66 + Alb [g/l] × (−0.0852). FIB-4 = (age [years] x AST [IU/L]) / (Plt [10^9^/L] x ALT [IU/L]^1/2^). APRI = (AST [IU/L] / upper limit of normal AST [IU/L]) x (100/platelet count [10^9^/L]). The Child-Pugh score was determined according to previously published methods [15].

### 2.4 CT Imaging

All patients underwent a four-phase CT examination of the abdomen using a dual-energy spectral-CT scanner (Revolution Apex, GE HealthCare) with 256-detector row. The contrast material (350 mgI/kg; Iomeron, Bracco, Italy) was administered at a rate of 4.0 mL/s, followed by a 30mL saline flush. Images of the arterial phase, portal phase, and equilibrium phase were obtained at 30 s, 60 s, and 180 s, respectively, after intravenous contrast material injection.

For the equilibrium phase, the dual-energy CT scan was performed using the following parameters: tube voltage, fast kVp switching (80 and 140 kVp); tube current, 360 mA; helical with pitch 0.984:1; tube rotation time, 0.8 s; detector coverage, 40 mm; DFOV, 25–30 cm, image reconstruction thickness 1.25 mm.

### 2.5 Extracellular Volume Fraction Analysis

**Fig. (2):**
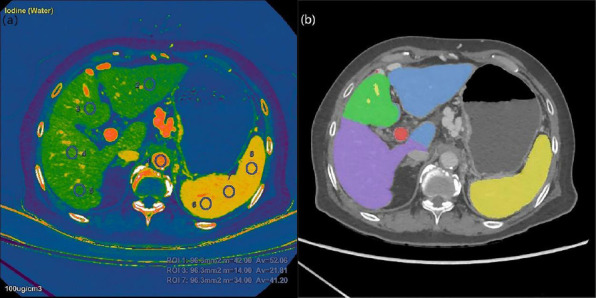
(**a**) ROIs measurement of quantitative indices based on Iodine (Water) images. (**b**) Liver and splenic volume measurements and liver segmentation were measured on a viewer workstation.

Two radiologists (7 and 4 years of experience in CT imaging) drew regions of interest (ROIs) on the iodine(water) images in a viewer workstation (AW 47, GE HealthCare). All ROIs were placed at three different levels (image reconstruction thickness 5 mm), with the liver hilar as the central one. For each slice of images, four ROIs (right anterior segment, right posterior segment, left lateral segment, and left medial segment) were placed in the liver parenchyma, three ROIs in the splenic parenchyma, and one ROI in the aorta (Fig. **2a**).

Quantitative indices of iodine concentration of the liver(L-IC), spleen(S-IC), and aorta(A-IC) were measured on the iodine(water) images, and normalized iodine concentration of the liver (L-NIC) and spleen (S-NIC) were calculated using the following formula: L-NIC=L-IC/A-IC, S-NIC=S-IC/A-IC. Spectral-CT derived ECV of liver (L-ECV) and spleen (S-ECV) were calculated using the following formula: L-ECV=(1-hematocrit)×L-NIC, S-ECV= (1-hematocrit)×S-NIC.

Volume-related parameters were measured by an abdominal radiologist with 7 years of experience on the portal phase images in AW47 workstation (Fig. **2b**). TLV was measured automatically. Liver segment separation was performed using software on an AW47 workstation semi-automatically. Liver segments II, III, and IV were separated by using the portal vein, liver segment I was separated by focusing on the unique anatomy. SV was measured manually.

### 2.6 Statistical Analyses

Patients were divided into the non-ACLD group, cACLD group, and dACLD group. Continuous variables were reported as mean and standard deviation for normally distributed data or the median and interquartile range for skewed data. One-way analysis of variance followed by an LSD test was used to compare normally distributed continuous data between multiple groups. Otherwise, a Kruskal-Wallis test and Mann-Whitney U with Bonferroni correction were applied. The area under the receiver operating characteristic curve (AUROCs) was calculated to evaluate the diagnostic performance of CT-related parameters (ECV, NIC, and CT volume) and non-invasive tests (including Child-Pugh score, ALBI, FIB-4, and APRI) for evaluating CLD severity. The DeLong test was adopted to compare the AUCs. The spearman correlations were performed to assess the correlation between non-invasive tests (including Child-Pugh score, ALBI, FIB-4, and APRI). The intraclass correlation coefficient (ICC) was used to assess interobserver reliability. Statistical analysis was performed using SPSS 20 (version 20.0.0) or MedCalc (version 20.027). A two-sided P-value<0.05 was considered statistically significant.

## 3 RESULTS

### 3.1 Baseline Characteristics

A total of 148 CLD patients [114 men and 34 women, aged 60 (51-69) years old; range 27-89 years old] were included in this study. The clinical baseline characteristics of the included patients are presented in Table **1**.

### 3.2 Comparison of Measurements between CLD Groups

The means of CT-related parameters in the dACLD, cACLD, and non-ACLD groups are shown in Table **2**. There were significant differences in L-ECV, S-ECV, L-NIC, S-NIC, TLV, LSVR, and SV among these groups (P<0.001). The L-ECV, S-ECV, L-NIC, S-NIC, and LSVR of dACLD groups were significantly higher than those of cACLD groups (P<0.05), and also significantly higher in cACLD groups compared to non-ACLD groups (P<0.05). The TLV of the dACLD group was significantly smaller than that of the cACLD group (P<0.05). The SV of the cACLD group was significantly larger than that of the non-ACLD group (P<0.05).

### 3.3 ROC of CT Parameters for Evaluating CLD Severity

**Fig. (3):**
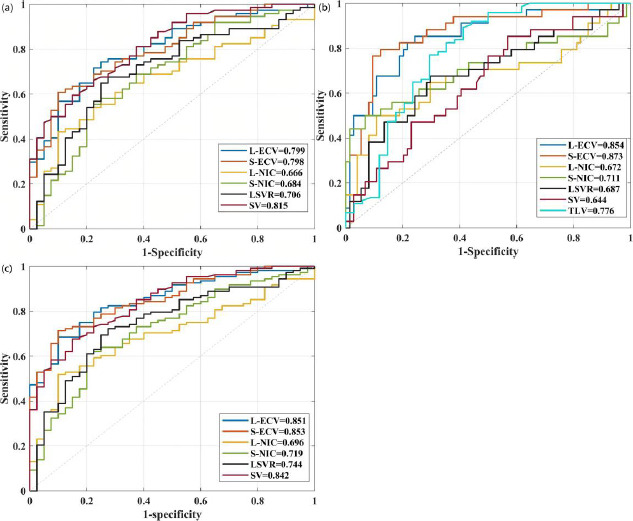
(**a**) The ROC curve of CT-related parameters in distinguishing between non-ACLD and cACLD. (**b**) The ROC curve of CT-related parameters in distinguishing between cACLD and dACLD. (**c**) The ROC curve of CT-related parameters in distinguishing between non-ACLD and ACLD.

The diagnostic performance of CT-related parameters in predicting CLD severity is shown in (Table **3** and Fig. **3a**-**c**). The ROC curve shows that SV exhibited the highest AUC in distinguishing non-ACLD and cACLD, followed by L-ECV and S-ECV, with AUC of 0.815, 0.799, and 0.797, respectively (all P<0.001). The S-ECV exhibited the highest AUC in distinguishing cACLD and dACLD, followed by L-ECV and TLV, with AUC of 0.873, 0.854, and 0.776, respectively (all P<0.001). The S-ECV exhibited the highest AUC in distinguishing non-ACLD and ACLD, followed by L-ECV and SV, with AUC of 0.854, 0.851, and 0.842, respectively (all P<0.001).

### 3.4 Correlation Analysis Between CT Parameters and Non-invasive Tests

**Fig. (4):**
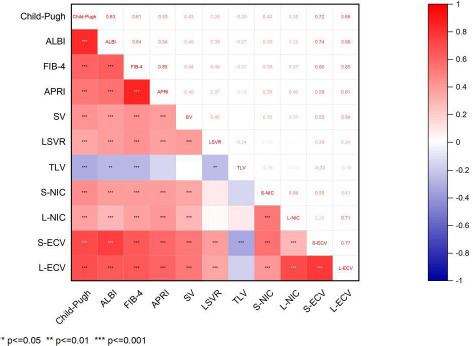
Correlation coefficient heatmap between CT-related parameters and non-invasive tests.

Spearman correlation analyses showed that L-ECV, S-ECV had stronger positive correlation with non-invasive tests (including Child-Pugh score [15], ALBI [16], FIB-4 [11], and APRI [4]). All correlations were statistically significant (P<0.001), and detailed results are presented in (Table **4** and Fig. **4**). The correlation coefficients between L-ECV and Child-Pugh, ALBI, FIB-4, and APRI are 0.693, 0.660, 0.652, and 0.607, respectively. For S-ECV, the correlation coefficients with Child-Pugh, ALBI, FIB-4, and APRI are 0.719, 0.742, 0.653, and 0.586, respectively.

### 3.5 The Comparison of the AUCs between CT-related Parameters and Non-invasive Tests

**Fig. (5):**
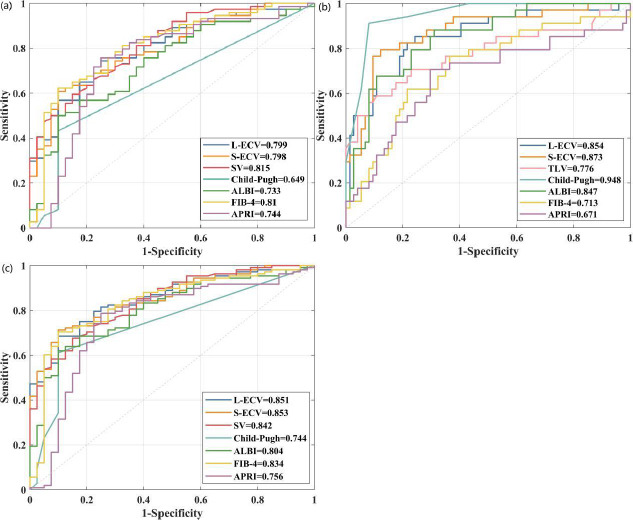
(**a**) The ROC curve of CT-related parameters and non-invasive tests in distinguishing between non-ACLD and cACLD. (**b**) The ROC curve of CT-related parameters and non-invasive tests in distinguishing between cACLD and dACLD. (**c**) The ROC curve of CT-related parameters and non-invasive tests in distinguishing between non-ACLD and ACLD.

The results of the ROC analysis showed that both CT-related parameters and non-invasive tests demonstrated the ability to predict CLD severity (all P<0.05). The SV demonstrated the highest AUC (0.815, P<0.001) in distinguishing between non-ACLD and cACLD (Fig. **5a**). Child-Pugh score exhibited the highest AUC (0.948, P<0.001) for differentiating between cACLD and dACLD (Fig. **5b**). S-ECV exhibited the highest AUC (0.853, P<0.001) for differentiating between non-ACLD and ACLD (Fig. **5c**). Moreover, liver and splenic ECV exhibit relatively high stability in assessing the severity of various stages of CLD.

In terms of the diagnostic performance between non-ACLD and cACLD, the DeLong test shows that the AUCs of SV, L-ECV, and S-ECV were higher than those of Child-Pugh (all P<0.05). Regarding the distinction between cACLD and dACLD, the AUCs of L-ECV and S-ECV were lower than those of Child-Pugh, but higher than those of FIB-4 and APRI (all P<0.05). The AUC of TLV was lower than that of Child-Pugh (P < 0.05). Regarding the distinction between non-ACLD and ACLD, AUCs of L-ECV and S-ECV were higher than those of Child-Pugh and APRI (all P<0.05). The AUC of SV was higher than that of Child-Pugh (P < 0.05).

### 3.6 Interobserver Agreement

The ICC for L-IC, S-IC, and A-IC measurements were 0.965 (P<0.001), 0.989 (P <0.001), and 0.970 (P<0.001), respectively. The ICC for cirrhosis imaging features was 0.902 (P<0.001). The ICC for moderate/severe ascites was 0.940 (P<0.001).

## 4 DISCUSSION

In our study, we evaluated different CT-related parameters to assess the severity in patients with CLD. The findings revealed that liver and splenic derived from spectral-CT demonstrated superior diagnostic performance in predicting the severity of CLD, as well as strongly correlated with non-invasive tests in these patients. To our knowledge, this is the first study utilizing liver and splenic ECV derived by spectral-CT for the severity stratification of CLD patients.

ECV is a physiologically intuitive measurement unit originally developed to quantify extracellular contrast agents in tissues, and it has been verified as a non-invasive measurement tool in assessing myocardial and liver fibrosis [17, 18]. Our results show that the liver and splenic ECV increases with the progression of CLD severity, being highest in dACLD. In early stages of CLD, excessive deposition of extracellular matrix (ECM) by hepatic stellate cells leads to fibrosis and disrupts the lobular structure of the liver [19]. As the disease progresses, necrotic areas form nodules, further altering liver architecture [9, 20]. These altered microenvironment and disrupted vascularity lead to the accumulation of CT contrast agents in the extracellular spaces of the liver. On the other hand, portal hypertension in CLD patients can lead to splenic congestion, which over time stimulates splenic tissue hyperplasia and fibrosis [21]. These pathological changes further affect the normal structure of the spleen, making it easier for extracellular CT contrast agents to accumulate. Consequently, the progression of CLD severity is reflected in the increased ECV in the liver and spleen, as demonstrated in our data.

The liver and splenic ECV assessment in our study was performed with the spectral-CT scanner. Spectral-CT, which utilizes a material decomposition algorithm, allows for more accurate evaluation of iodine concentration in the tissue [22]. However, the differences between spectral-CT ECV and NIC in CLD patients remain unclear. The results of this study indicate that the diagnostic performance of liver and splenic NIC is relatively low when evaluating the severity of CLD. NIC primarily reflects tissue blood flow, but iodine concentration measurement may be affected by various factors such as the rate of contrast agent injection and individual differences in blood circulation [20]. In contrast, liver and splenic ECV can directly reflect the pathological changes in the extracellular space, which may result in superior performance in the risk stratification of CLD patients.

Our research demonstrated that splenic volume is an effective parameter for identifying patients with ACLD and cACLD, with AUCs of 0.842 and 0.815, respectively. Splenomegaly serves as a critical sign of portal hypertension in patients with CLD, caused by the back pressure from the congested portal venous system [23-25]. And the result of our study suggested that splenic volume seems to be an important indicator in predicting early ACLD. According to our study’s investigation, the total liver volume of CLD patients does not significantly change before the development of hepatic decompensation. Mario's [26] study demonstrated significant atrophy of the whole liver occurs during the decompensated phase of cirrhosis, which is analogous to our research findings. This stability in liver volume during compensated phases of CLD may reflect the liver's inherent capacity for regeneration and its ability to adapt to the disease process.

We also investigated the correlations between ECV and non-invasive tests. The results indicated that liver ECV demonstrated stronger correlations with FIB-4 (r=0.653, p<0.001) and APRI (r=0.607, p<0.001). Studies have shown that increased liver ECV directly reflects abnormal accumulation of ECM within the liver [18], which could be the underlying reason for its higher correlation with these fibrosis indices. On the other hand, splenic ECV showed stronger correlations with Child-Pugh score (r=0.719, p<0.001) and ALBI (r=0.742, p<0.001). Notably, elevated splenic ECV, which is considered an imaging marker of portal hypertension [27, 28], may explain the link between splenic ECV and liver function indicators. Non-invasive tests such as Child-Pugh classification and FIB-4 are widely utilized for assessing the liver function and predicting the prognosis of CLD [29, 30]. Nevertheless, as far as we know, a systematic comparison among liver and splenic ECV, CT volume, and non-invasive tests in severity stratification of CLD has not been reported yet. Compared with CT volume and non-invasive tests, ECV exhibits relatively high stability in assessing the severity of various stages of CLD. The assessment of spectral-CT liver and splenic ECV can provide supplementary information for the pathological changes in CLD and offer a nuanced understanding for clinical assessment of disease progression.

## LIMITATION

There were several limitations to our study. Firstly, the sample size in our study was relatively small, particularly for patients with dACLD. This limitation could have introduced bias and limited the generalizability of our findings. Secondly, the grouping of CLD patients in our study was mainly based on clinical histories and relevant previous imaging data, while histological confirmation was not obtained. However, liver biopsy has become less commonly indicated as the significant advancements in the field of non-invasive tests over the past decades, which reflects current clinical practice. Thirdly, the causes of CLD in our study included hepatitis B, hepatitis C, alcohol-related liver disease, and autoimmune liver disease, with hepatitis B patients in the majority. It remains unclear whether the ECV would vary based on the specific etiology of CLD. Further investigation with a larger sample size and more detailed analysis is required to ensure the generalizability of the findings to different etiologies of CLD.

## CONCLUSION

In conclusion, spectral-CT liver and splenic ECV can effectively reflect the dynamic progression of CLD and correlate well with non-invasive tests in these patients. Spectral-CT liver and splenic ECV can serve as non-invasive imaging biomarkers for severity stratification, without the need for additional costs. For CLD patients with higher spectral-CT liver and splenic ECV, indicating more advanced disease, they may be prioritized for more aggressive therapies or closer follow-up. In the future, large-scale, multi-center studies are needed to establish its clinical utility and validate its predictive value for prognosis.

## Figures and Tables

**Table 1:** The clinical baseline characteristics of the CLD patients.

**Characteristic**	**CLD Patients (n=148)**
General features	-
Age (years)	60 (51-69)
Sex (M:F)	114:34
Etiology for CLD	-
Hepatitis B virus	103 (69.59%)
Hepatitis C virus	11 (7.43%)
Alcoholic liver disease	23 (15.54%)
Autoimmune liver disease	11 (7.43%)

**Table 2:** Comparisons of ECV, NIC and CT volume-related parameters between CLD groups.

-	**dACLD (n=34)**	**cACLD (n=74)**	**non-ACLD (n=40)**	**P (dACLD *vs* cACLD)**	**P (cACLD *vs* non-ACLD)**
L-ECV	45.26±8.04	33.93(31.03-37.99)	29.54±3.48	<0.001	<0.001
S-ECV	53.85±7.36	41.41(38.18-45.80)	36.93±3.34	<0.001	<0.001
L-NIC	0.63±0.11	0.56±0.08	0.52±0.06	<0.001	0.012
S-NIC	0.74±0.10	0.68±0.05	0.65±0.05	<0.001	0.017
TLV	802(677-1074)	1221(1005-1397)	1185(1096-1328)	<0.001	1.000
LSVR	88.97±34.58	64.50(50.00-81.00)	48.50(35.50-58.00)	0.014	0.002
SV	519(336-928)	345(224-602)	177(136-256)	0.096	<0.001

**Table 3:** The ROC curve of ECV, NIC, and CT volume-related parameters for evaluating CLD severity.

-	**AUC**	**P**	**Cut-Off**	**Sensitivity(%)**	**Specificity(%)**	**95% CI**
**non-ACLD *vs* cACLD**						
L-ECV	0.799	<0.001	31.23	74.32	75.00	[0.713, 0.868]
S-ECV	0.797	<0.001	39.62	60.81	90.00	[0.712, 0.867]
L-NIC	0.668	0.001	0.57	43.24	90.00	[0.574, 0.753]
S-NIC	0.683	0.001	0.67	54.05	77.50	[0.589, 0.767]
LSVR	0.706	<0.001	54.92	67.57	72.50	[0.613, 0.787]
SV	0.815	<0.001	282	59.46	85.00	[0.731, 0.882]
**cACLD *vs* dACLD**						
L-ECV	0.854	<0.001	37.89	85.29	75.68	[0.773, 0.914]
S-ECV	0.873	<0.001	49.63	79.41	87.84	[0.795, 0.929]
L-NIC	0.671	0.007	0.65	50.00	89.19	[0.574, 0.759]
S-NIC	0.709	0.001	0.74	52.94	87.84	[0.614, 0.792]
TLV	0.776	<0.001	835	58.82	89.19	[0.686, 0.851]
LSVR	0.687	0.001	75.23	67.65	70.27	[0.591, 0.773]
SV	0.644	0.011	285	85.29	43.24	[0.547, 0.734]
**non-ACLD *vs* ACLD**						
L-ECV	0.851	<0.001	32.93	68.52	90.00	[0.784, 0.904]
S-ECV	0.852	<0.001	39.62	71.30	90.00	[0.785, 0.905]
L-NIC	0.698	<0.001	0.57	51.85	90.00	[0.617, 0.771]
S-NIC	0.719	<0.001	0.67	61.11	77.50	[0.639, 0.789]
LSVR	0.744	<0.001	54.92	72.22	72.50	[0.666, 0.812]
SV	0.842	<0.001	282	67.59	85.00	[0.773, 0.897]

**Table 4:** Correlation coefficient between CT-related parameters and non-invasive tests.

-	**Child-Pugh**	**ALBI**	**FIB-4**	**APRI**
L-ECV	0.693***	0.660***	0.653***	0.607***
S-ECV	0.719***	0.742***	0.652***	0.586***
L-NIC	0.378***	0.294***	0.373***	0.404***
S-NIC	0.443***	0.381***	0.383***	0.388***
TLV	-0.305***	-0.266**	-0.274***	-
LSVR	0.357***	0.389***	0.436***	0.368***
SV	0.426***	0.400***	0.435***	0.399***

**Note:** ***P<0.001; **P<0.01; *P<0.05

## Data Availability

All data generated or analyzed during this study are included in this published article.

## LIST OF ABBREVIATIONS

CLD: Chronic liver diseas
non-ACLD: Non-advanced chronic liver disease
cACLD: Compensated advanced chronic liver disease
dACLD: Decompensated advanced chronic liver disease
spectral-CT: spectral computed tomography
L-ECV: Extracellular volume fraction of liver
S-ECV: Extracellular volume fraction of spleen
L-NIC: Normalized iodine concentration of liver
S-NIC: Normalized iodine concentration of spleen
TLV: Total liver volume
LSVR: Liver segmental volume ratio
SV: Splenic volume
ALBI: Albumin-bilirubin Index
FIB-4: Fibrosis Index based on the 4 factors
APRI: Aminotransferase-to-platelet ratio Index

## STANDARDS OF REPORTING

STROBE guidelines were followed.
